# Mechanical Properties and Wear Resistance of Sulfonated Graphene/Waterborne Polyurethane Composites Prepared by In Situ Method

**DOI:** 10.3390/polym10010075

**Published:** 2018-01-15

**Authors:** Jianyan Feng, Xuechuan Wang, Peiying Guo, Yujie Wang, Xiaomin Luo

**Affiliations:** 1 College of Bioresources Chemical and Materials Engineering, Shanxi University of Science & Technology, Xi’an 710021, Shanxi, China; fengjianyan2008@163.com (J.F.); m17612082503@163.com (Y.W.); luoxiaomin@sust.edu.cn (X.L.); 2 National Demonstration Center for Experimental Light Chemistry Engineering Education, Shanxi University of Science & Technology, Xi’an 710021, Shanxi, China; 3 School of Arts and Sciences, Shanxi University of Science & Technology, Xi’an 710021, Shanxi, China; guopy@sust.edu.cn

**Keywords:** sulfonated grapheme, waterborne polyurethane, in situ method, mechanical resistance properties, wear resistance

## Abstract

In order to improve the dispensability of graphene oxide (GO) in waterborne polyurethane (WPU), sulfonated graphene (SGO) with superior dispersity was prepared by modifying graphene oxide with sodium 2-chloroethane sulfonate to introduce hydrophilic sulfonic groups into the structure. SGO/WPU composites were prepared using isophorone diisocyanate (IPDI), polytetramethylene ether glycol (PTMEG 2000), dimethylolpropionic acid (DMPA) and SGO as raw materials. The influence of SGO content on composite properties were investigated. The structure and morphology of SGO and SGO/WPU composites were characterized by infrared spectroscopy, X-ray diffractometry and transmission electron microscopy etc. Their mechanical properties and wear resistance were analyzed as well. The experimental results showed that SGO was successfully grafted onto polyurethane macromolecule by an in situ method and, with the introduction of sulfonic groups, the interfacial compatibility of GO and PU was improved significantly so that SGO evenly dispersed into WPU. The SGO that was grafted onto WPU macromolecules exhibited layered morphology with nanometers in the WPU matrix. With increasing SGO content, the tensile strength and the wear resistance of the film increased, but the addition of more than 0.8 wt % SGO yielded unfavorable results. When the added amount of SGO was 0.8 wt % of WPU, the tensile strength of the composite film was 46.53% higher than that of the blank group, and the wear resistance of the film was remarkably improved, which was due to a strong interaction between the SGO and WPU phases. Thus, the conclusion can be drawn that appropriate amount of SGO addition can enhance the mechanical properties of SGO/WPU composite film.

## 1. Introduction

Since the discovery of preparing graphene (G) by micromechanical stripping in 2004, its extraordinary physical and chemical properties were identified, thus increasing the enthusiasm of researchers [1,2]. Owing to its unique two-dimensional structure, grapheme exhibits much better properties than traditional inorganic materials, resulting in its extensive application in functional materials [3,4,5,6]. However, because of the absence of functional groups on its surface and a relatively strong interaction of van der Waals force and π–π stacking between its layers, grapheme aggregates easily in solution or polymer [7,8]. Graphene Oxide (GO), a kind of derivative, is compatible with polymer after modification because there is large amount of epoxy, hydroxyl and carboxyl on its surface [9,10]. As a result, G is hydrophobic in nature whereas GO is hydrophilic, i.e., easily dispersible in water. However, the quality of the produced graphene is not suitable for electronic applications or mechanical reinforcement of polymers due to structural defects created during the synthesis of GO [11]. Still, this is a preferable route for large-scale handling of graphenic materials with tailored surface properties by functionalization. Zhang et al. [12] successfully grafted octadecylamine onto GO surface through amidation and nucleophilic substitution reaction with carboxyl (–COOH) and epoxy (–O–) to obtain G-ODA nanocomposites. The introduction of octadecylamine increased the compatibility with organic solvents, which made it possible for GO to fully disperse in chloroform. Another example is the successful functionalization of the covalent bond of different natural amino acid molecules with GO sheets by Mallakpour et al. [13]. The functionalized GO not only disperses well in water and common organic solvents, but also exhibits nonlinear optical property. In view of the high hydrophilicity of sulfonic acid groups, GO can be sulfonated, through which its dispersibility is improved while its original properties are remained. So, sulfonation has become one of the research focuses on carbon material modification. The sulfonated grapheme oxide (SGO) was modified by grafting sulfonic acid groups onto GO terminal groups [14,15]. The modified SGO can facilitate the uniformity of graphene aqueous solution, making it exhibiting excellent physicochemical properties in aqueous solution or organic solution.

Waterborne Polyurethane (WPU) is non-toxic, non-flammable, odorless and pollution free. With such unusual properties, WPU coating is an ideal alternative material of the traditional solvent polyurethane, and therefore has been widely applied to various fields such as fabric coating, synthetic leather and natural leather coating [16,17]. The properties of most pristine WPUs are poorer than those of solvent-based PUs, which include a softer texture, poorer electrical insulation, wear-resistance and mechanical property. Therefore, improving electrical insulation, tensile strength and wear-resistance is meaningful for the development of WPU. Their properties can be improved by many methods. A common method is adding cross-linking agents as UV-curable compounds [18,19]. Another method is forming organic and inorganic hybrids by introducing inorganic fillers as carbon nanotubes, clays, or graphenes to WPUs [20,21,22,23]. Graphene is acrystalline allotropeof carbon with a 2-dimensional nanostructure. It presents unique properties such as high hardness, electrical conductivity and heat conduction. When it is used to modify WPU, the coating materials will exhibit high physical properties and functions such as wear and scratch resistance, static resistance, electricity and heat conductivity, flame retardancy and electromagnetic shielding, thereby improving the quality of fabric coating and leather [24,25,26]. Choi et al. [27] used an appropriate amount of sodium dodecyl sulfate in water and sonicated to get a colloidal aqueous dispersion of FGS, then nanocomposites of waterborne polyurethane (WPU) reinforced with functionalized graphene sheets (FGSs) were effectively prepared by casting from a colloidal dispersion of FGS/WPU and the morphology and physical properties were examined. As a result, improved conductivity and thermal resistance by FGS were more evident with finer FGS dispersion in the nanocomposites. Sun et al. [28] prepared composite coatings by mixing graphene and polyurethane. It was found that the thermal, mechanical and wear-resistance properties of the coating had been significantly improved with the addition of graphene. Both solvent resistance and tensile strength increased first then decreased, but tensile strength reached the maximum when the added content was 0.1 wt %. Suen et al. [29] synthesized waterborne graphene oxide/poly (siloxane-urethane) nanocomposites by an in situ method. With increasing GO content, the average particle size, viscosity, and ionic conductivity of the GO/SWPU dispersion increased; adding GO improved the thermal and mechanical properties of the GO/SWPU nanocomposites.

In this study, sulfonated graphene oxide (SGO) was prepared by modifying graphene oxide (GO) using sodium 2-chloroethanesulfonate monohydrate. SGO dispersion, excellent in aqueous solution with hydrophilic sulfonic acid groups, were introduced to GO. In addition, waterborne polyurethane pre-polymer (NCOPU) was prepared using isophorone diisocyanate (IPDI), polytetramethylene ether glycol (PTMEG 2000) and dimethylolpropionic acid (DMPA). Then, SGO/WPU composite materials were synthesized through SGO and NCOPU in situ method. SGO was used to improve the wear resistance and mechanical properties of waterborne polyurethane film.

## 2. Experimental

### 2.1. Materials

Oxidized graphite SE2430(CP) was obtained from The Sixth Element (Changzhou) Materials Technology Co., Ltd. (Changzhou, China). Isophorone diisocyanate (IPDI, 99%), dimethylolpropionic acid (DMPA, 98%), sodium 2-chloroethane sulfonate (ClCH_2_CH_2_SO_3_Na, 98%), triethylamine (TEA, 99%) and dibutyltin dilaurate (DBTDL, 98%),acetone (AR) and absoluteethylalcohol were purchased from Aladdin Biological Technology Co., Ltd. (Shanghai, China). In addition, polytetramethylen etherglycol (PTMEG 2000) was obtained from Huada Chemical Group Co., Ltd. (Yantai, China).

### 2.2. Preparation of SGO

400 mg of oxidized graphite SE2430 were added into 400 mL of deionized water under ultrasonication condition for 1 h prepared oxidized graphene dispersion. 5 g of sodium 2-chloroethane sulfonate (ClCH_2_CH_2_SO_3_Na, 98%) and 1.2 g of NaOH were added into the graphene oxide dispersion under ultrasonication and 35 °C condition continue to reaction for 3 h and then 1.5 mL of HNO_3_ was added into the mixture. Finally, the mixture was washed with ethanol three times and put into vacuum drier for 2 days to achieve the dry SGO [14].

### 2.3. Preparation of SGO/WPU

15.6 g polytetramethylene ether glycol (PTMEG 2000), 4.4 g isophorone diisocyanate (IPDI) and small dibutyltin dilaurate (DBTDL, 98%) were added to the reaction bottle. Then, the reaction mixture was stirred mechanically at 200 rpm and 85 °C for 2 h in the prepared polyurethane pre-polymer (NCOPU). The dimethylolpropionic acid (DMPA) was added into polyurethane pre-polymer for chain extension reaction at 85 °C for 1 h, then SGO acetone dispersion was added (the SGO of 0.8 wt % of the pre-polymer was sonicated in a certain amount of acetone, the SGO concentration was 2 mg/mL).The chain extension reaction was allowed to proceed for 2 h at 80 °C to obtain SGO/WPU polymer. The polymer was neutralized by triethylamine at 50 °C and stirred at 300 rpm for 30 min to become a SGO/WPU anionomer solution. Distilled water was added to the anionomer solution at 1000 rpm for 30 min, and then acetone was distilled off under reduced pressure to obtain aqueous SGO/WPU dispersions with a solid content of 30 wt %. The symbols WPU-0, SGO/WPU-1, SGO/WPU-2, SGO/WPU-3, and SGO/WPU-4, represent the SGO containing 0.0, 0.4, 0.6, 0.8, and 1.0 wt % of NCOPU, as recorded in Table 1.

### 2.4. Preparation of SGO/WPU Composite Film

The SGO/WPU films can be prepared from the nanocomposite dispersions. 5.0 g SGO/WPU dispersion was added into the Teflon plate mold fully dried for 48 h at 50 °C. The symbols WPU-0, SGO/WPU-1, SGO/WPU-2, SGO/SWPU-3 and SGO/WPU-4 are shown in Table 1.

**Figure 1:**
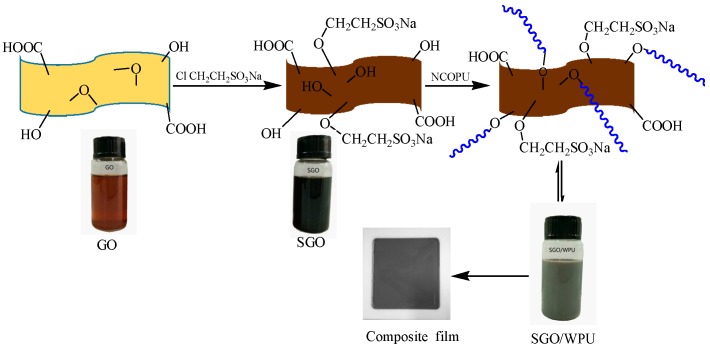
Schematic diagram about the synthesis route for SGO/WPU.

**Figure 2:**
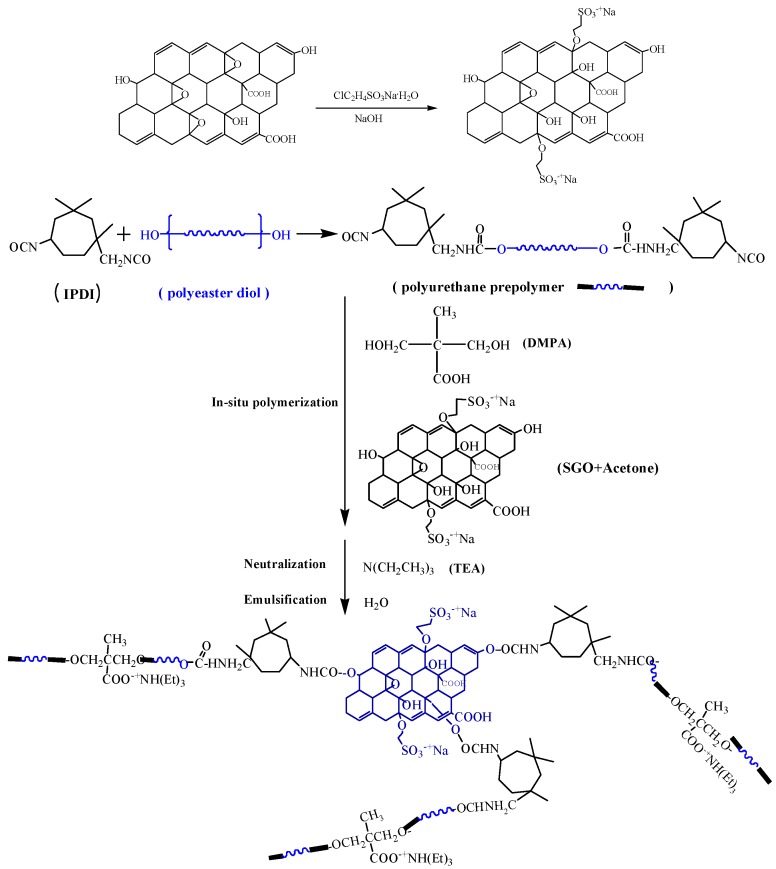
Schematic diagrams of sulfonated modified reaction of GO and synthesis of SGO/WPU.

Figure 1 and Figure 2 show the procedure for preparing SGO/WPU and their reaction mechanism.

### 2.5. Characterization

FTIR bromide tabletting was used to characterize the chemical structure of GO, SGO and SGO/WPU on a VECTOR-22 FTIR instrument (Thermo Scientific, Waltham, MA, USA). FTIR spectra were collected in the range 4000~400 cm^−1^. X-ray photoelectron spectroscopy (XPS) was carried out with AXIS SUPRA spectrometer (Kratos, Manchester, UK), using Al Kα excitation radiation. X-ray diffraction (XRD) was measured using a D/max2200PC X-raydiffractometer (Bruker AXS, Billerica, MA, USA) with Cu Kα radiation at 40 kV and 50 mA. Data were obtained in the 2θ angle of 5~60° with the scanning rate of 5°/min. Transmission electron microscopy (TEM, Hitachi, model H7650, Tokyo, Japan) was used to examine the morphology of the GO and SGO. The samples for TEM examination were prepared by placing a few drops of dispersion onto a lacey carbon film support on a Cu grid. Images were acquired using a transmission electron microscope operated at 80 kV. Thermogravimetric analysis (TGA) was performed using STA449F3 TGA (Netzsch, Bavaria, Germany) and the samples (5~8 mg) were heated from room temperature to 600 °C under nitrogen at a rate of 10 °C/min.

Tensile strength and elongation at break were measured using a universal testing machine; model TH-8203S (Tuobo, Suzhou, China). Testing was conducted based on ASTM D2209-1995 with 50 N load and 100 mm/min tensile rate. Wear resistance was tested using DZ-204 (Dazhong, Dongguan, China) Wear Testing Machine. Each sample was cut into a circle with a diameter of 12 cm with the test condition of 500 g load and 200 laps.

## 3. Results and Discussion

### 3.1. The Structure and Morphology of GO and SGO

**Figure 3:**
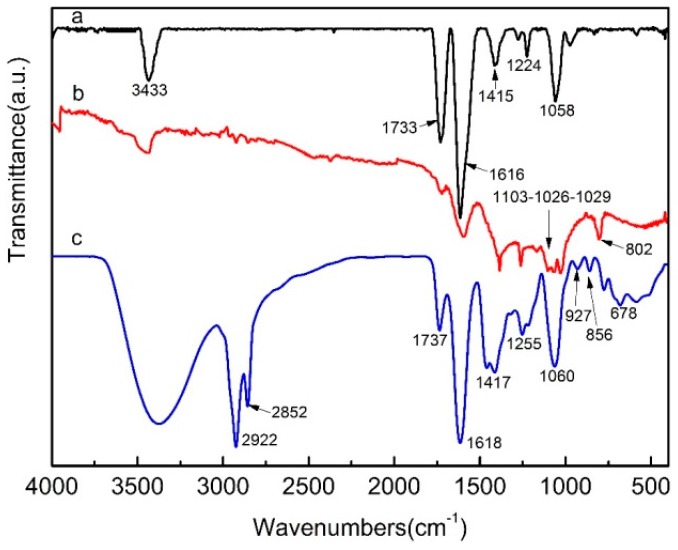
FTIR spectra of the GO (a), SGO (b) and SGO/WPU-3 (c).

The FTIR spectra of the GO (a), SGO (b) and SGO/WPU-3 (c) are shown in Figure 3. From the GO (a) spectrum curve, it can be observed that –OH stretching vibration peak appeared near 3433 cm^−1^, C=O stretching vibration peak appeared at 1733 cm^−1^, C–OH bending vibration peak appeared at 1616 cm^−1^, C–O stretching vibration peak of carboxyl appeared at 1415 cm^−1^ and C–O–C vibration absorption peak of epoxy group appeared at 1058 cm^−1^. There is a clear decrease in C–OH stretching vibration peak of the modified SGO at 3433 cm^−1^ and 1616 cm^−1^, and C–O–C vibration absorption peak of epoxy group at 1058 cm^−1^ practically disappeared. Simultaneously, three absorption peaks of –S=O deformation vibration appeared at 1103 cm^−1^, 1026 cm^−1^ and 1029 cm^−1^ respectively and–CH_2_ plane swing absorption vibration appeared in 802 cm^−1^, suggesting that sodium 2-chloroethane sulfonate monohydrate had successfully modified GO and sulfo group was grafted onto GO. As shown in SGO/WPU-3 (c), –NH stretching vibration at 3350 cm^−1^ increased significantly; the peaks at 2922 cm^−1^ and 2852 cm^−1^ are stretching vibration peaks of –CH_3_, –CH_2_. The stretching vibration peaks of C=O in amide bond at 1737 cm^−1^ increased. The stretching vibration peaks of –C–O in carbamate appeared at 1255 cm^−1^, the stretching vibration peaks of –C–O at 1060 cm^−1^and deformation vibration coincidence peak of –S=O in ether group increased significantly. These phenomena indicated that SGO/WPU had been successfully prepared.

**Figure 4:**
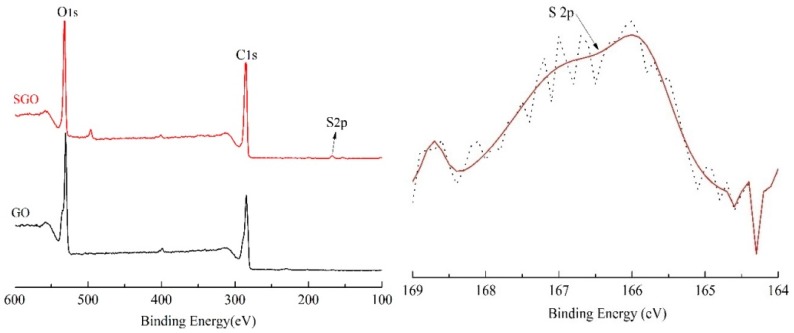
XPS spectra of SGO.

Figure 4 shows XPS spectra of GO and SGO. By analyzing the full scan spectra of GO and SGO, we found that the C/O values of GO and SGO were 2.18 and 2.81 respectively. Evidently, the content of oxygen in GO is higher than that of SGO, suggesting that the structure of GO has a certain degree of reduction during sulfonation modification. The S2p characteristic absorption peak appeared in 167.5 eV, which is attributed tothe introduction of –SO_3_^−^ in the reaction between graphene oxide and sodium 2-chloroethane sulfonate indicating the existence of SGO [14].

**Figure 5:**
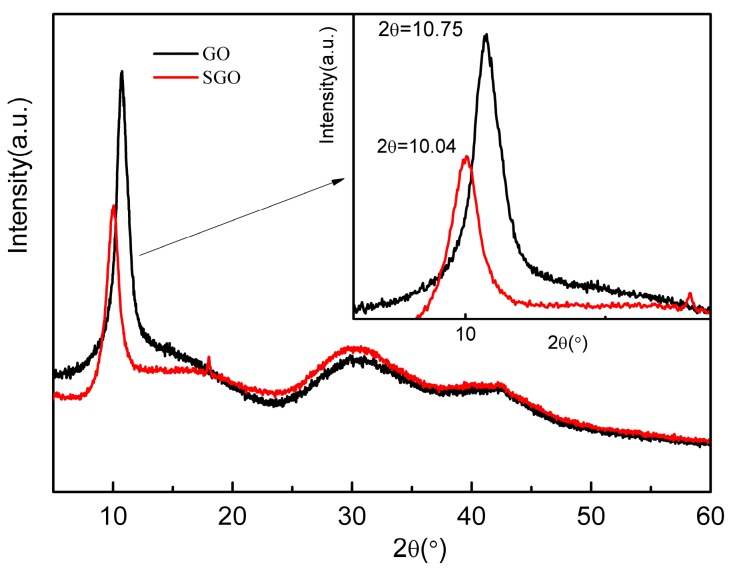
X-ray diffraction spectra of GO and SGO.

The XRD map of GO and SGO is shown in Figure 5. A sharp and narrow diffraction peak of GO appeared at 10.75°. According to the Prague equation, the spacing between GO layers was calculated to be 0.822 nm, showing that the oxygen functional groups such as –COOH, –OH, –O– on GO layers reduced the van der Waals force between the layers. Meanwhile, because of the steric effect among groups, van der Waals force between the GO layers was further weakened, consequently, increasing the distance between layers. SGO has obvious diffraction peaks around 10.04° (d = 0.879 nm). With the introduction of sulfonic acid groups in the GO layer through sulfonation reaction, the interlayer spacing in GO layers was increased. The electrostatic repulsion and GO crystallization was destroyed, leading to the increase of structure disorder [30].

**Figure 6:**
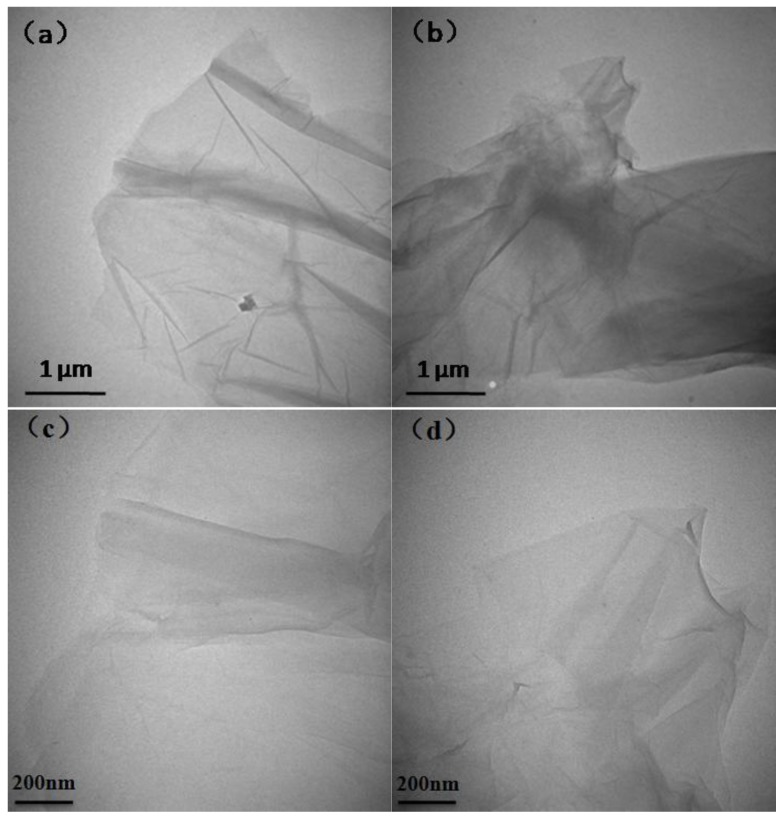
TEM microphotographs of GO (**a**,**c**) and SGO (**b**,**d**).

Figure 6 shows electron microscopic photographs analysis of GO and SGO TEM. From Figure 6, it can be clearly seen that the GO and SGO obtained in the experiment are of a semitransparent chiffon-like lamellar structure with obvious folds. The material has a large specific surface area against the thickness of the lamellar. As is shown Figure 6b, there are more wrinkles at the edge of SGO, indicating that the sodium 2-chloroethane sulfonate was grafted onto GO surface through covalent modification. This morphology might reduce the SGO lamellar surface energy, making SGO be in stable existence. SGO shell structure can be clearly noted from the edge of Figure 6d. 

### 3.2. The Structure and Morphology of SGO/WPU

**Figure 7:**
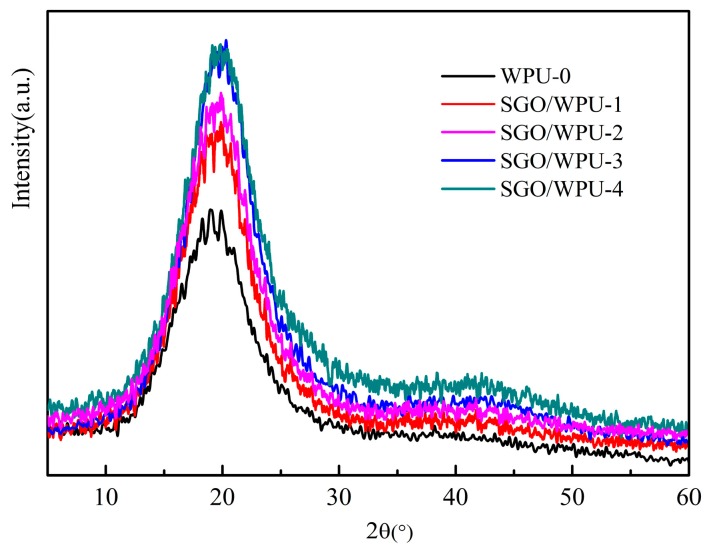
X-ray diffraction spectra of WPU and SGO/WPU.

The XRD maps of WPU-0, SGO/WPU-1, SGO/WPU-2, SGO/WPU-3 and SGO/WPU-4 are shown in Figure 7. The XRD patterns showed same characteristics for all SGO/WPU nanocomposites. The broad peaks at 20° are attributed to the amorphous phases in polyurethane. With the addition of SGO, no shape or narrow peaks associated with the crystalline phase the composite films diffraction peaks broadened slightly [28]. In addition, SGO in situ grafted onto WPU macromolecules as nano reinforcing material was fully bonded with the two-phase interface of polyurethane in drying process of the film due to the small amount of SGO addition; the influence on the crystallinity of polyurethane is not obvious.

**Figure 8:**
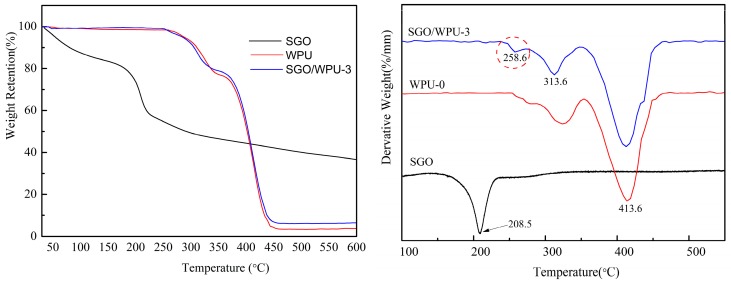
TGA curves of the SGO, WPU and SGO/WPU nanocomposites.

Figure 8 shows the decomposition peak temperatures and the temperature at maximum thermal decomposition rate, respectively. The maximum thermal decomposition rate of SGO was at 208.5 °C. With the added amount of SGO, new degradation peaks at maximum thermal decomposition rate appeared in SGO/WPU. Compared with the SGO curve and WPU-0 curve, the SGO/WPU-3 exhibited new degradation peaks at 258.6 °C, which further indicated that SGO was successfully grafted onto WPU. 

**Figure 9:**
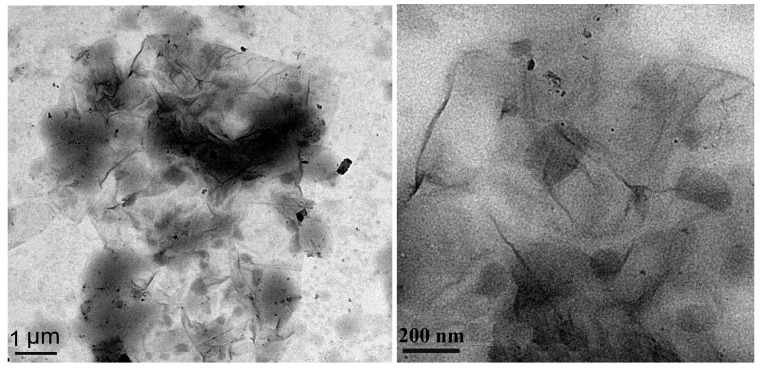
TEM microphotographs of SGO/WPU.

Figure 9 shows the TEM microphotograph of the SGO/WPU-3 nanocomposite. The dark lines are SGO sheets and the light-colored parts are polyurethane molecule. The SGO grafted on WPU macromolecule exhibited layered morphology with nanometer in the WPU matrix.

### 3.3. Mechanical Properties of SGO/WPU Composites

**Figure 10:**
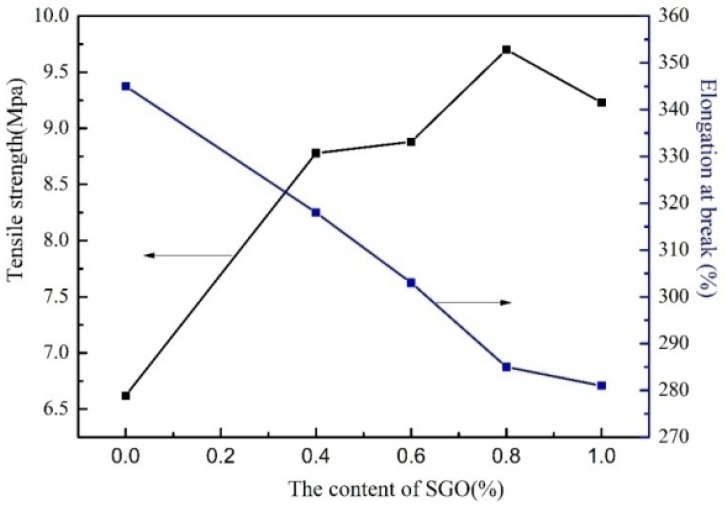
Mechanical properties of SGO/WPU composites with different SGO contents.

Table 2 and Figure 10 shows the testing results of the mechanical properties of SGO/WPU composites with different SGO content.

As is shown in Table 2 and Figure 10, with the addition of SGO, the tensile strength of SGO/WPU composite film increased significantly and then decreased. When the added content of SGO was 0.8 wt %, the tensile strength of the composite film reached 9.70 MPa, which is 46.53% higher than that of the blank film. This is because that when polyurethane molecules were formed by the in situ reaction of SGO and polyurethane pre-polymer as shown in Figure 2 and Figure 9, SGO was grafted onto polyurethane macromolecules as sulfonate chain extender. Then, a new hard segment structure was formed through the reaction of –OH and –NCO in SGO, so the rigidity of the material was improved. Meanwhile, the introduction of sulfonic acid-based hydrophilic groups improved the interfacial compatibility between graphene and polyurethane, leading to the uniform dispersion of SGO in aqueous polyurethane. However, with the addition of SGO, SGO and DMPA competed to react with pre-polymer NCO, affecting the chain extension reaction between pre-polymer and DMPA. As a result, the average molecular weight of polyurethane was affected and some SGO molecules bonded with polyurethane molecules in the form of molecular hydrogen bonds; therefore, tensile strength of the obtained composite films were reduced. When SGO, as a hard segment, was grafted onto polyurethane molecules, the tensile strength of the SGO/WPU composite film would increase, but the toughness of the composite film were weakened to some extent. With the increasing SGO content in the composite film, the elongation at breaking rate was obviously reduced. That is, the more SGO content in the composite film, the weaker the toughness.

### 3.4. Abrasion Resistance Test

**Figure 11:**
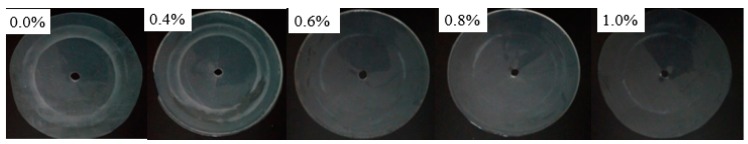
Stand wears and tears of SGO/WPU composites with different SGO contents.

Table 3 and Figure 11 show the result of abrasion resistance test.

**Figure 12:**
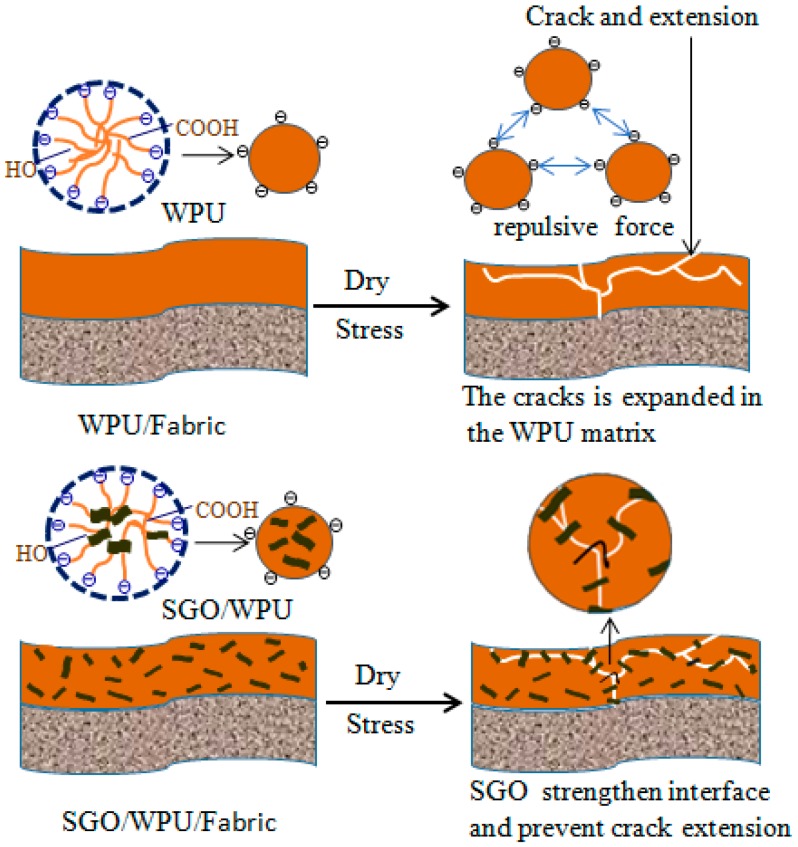
Schematic diagram of SGO enhanced wear-resistant.

It can be seen from Table 3 and Figure 10 that pure water polyurethane film exhibits the poorest wear resistance. With the addition of SGO, its wear resistance increased, indicating that the addition of SGO is helpful to improve the wear resistance of the film. As is shown in Figure 11, when the added SGO content was 0.8 wt %, almost no damage was found in the film when the wheel was turned 200 times, which also shows that SGO can help to enhance wear resistance of WPU membrane materials. The enhanced wear resistance mechanism is shown in Figure 12. A pure anion WPU was influenced by electrostatic repulsion during the drying process, and cracks can easily occur in the polyurethane matrix. With the influence of stress, cracks finally become destructive fractures. When SGO was grafted onto the polyurethane macromolecules by in situ, the hard segment ratio increased, SGO not only distributed uniformly in WPU, but had a covalent and hydrogen bond with the polyurethane molecules, which significantly improved the combination of SGO and PU and reduced the stress accumulation point. In this way, the crack extension WPU caused by stress was reduced and the occurrence of destructive fracture can be prevented. 

With the addition of SGO content, the specific surface area of nanomaterials increased, and the contact area with waterborne polyurethane increased as well. More cracks were produced when the material was subjected to stress impact. As a result, the absorption effect of the impact energy increased, so the possibility of damaging cracks was reduced. Furthermore, as SGO has higher hardness, the modified polyurethane with SGO can significantly improve the hardness of composite films, and, consequently, the wear resistance of the film can be improved as well.

## 4. Conclusions

In summary, SGO was prepared by sodium 2-chloroethane sulfonate by the reaction with epoxy group in alkaline conditions. The introduction of –SO_3_^−^ groups on the GO surface by covalent modification was proved by FTIR and XPS, indicating that GO lattice structure was destroyed by sulfonated modification and the structure of disorder was further increased. SGO/WPU composites were prepared by an in situ method to graft SGO onto WPU macromolecules; SGO layer structure with thickness in the nano-scale; SGO was grafted onto WPU macromolecules and exhibits layered morphology with nanometer in; the WPU matrix was verified by TEM. With increasing SGO content, the tensile strength and wear resistance of the film increased, but adding more than 0.8 wt % SGO yielded unfavorable results, which were possibly caused by the agglutination of SGO in WPU. When the added SGO content was 0.8 wt % of WPU, the tensile strength of the composite film increased by 46.53%; the interfacial compatibility of SGO and WPU was significantly improved by introducing hydrophilic sulfonic groups into the SGO/WPU composites. As a result, SGO was uniformly dispersed into WPU, and generated strong interaction between the SGO and WPU phases. Thus, the conclusion can be drawn that adding an appropriate amount of SGO can improve the wear-resistance of composite films significantly. 

## Figures and Tables

**Table 1:** Recipes for preparation of different SGO/WPU dispersion.

Samples	m(NCOPU)/g	V(SGO Acetone Solution)/mL	SGO/wt %
WPU-0	20	0	0.0
SGO/WPU-1	20	40	0.4
SGO/WPU-2	20	60	0.6
SGO/WPU-3	20	80	0.8
SGO/WPU-4	20	100	1.0

**Table 2:** Mechanical properties of SGO/WPU composites with different SGO contents.

Sample	Elongation at Break/%	Tensile Strength/MPa	Young Modulus/MPa
WPU-0	355	6.62	0.18
SGO/WPU-1	318	8.78	0.22
SGO/WPU-2	303	8.89	0.23
SGO/WPU-3	287	9.70	0.25
SGO/WPU-4	285	9.33	0.24

**Table 3:** Abrasion resistance of SGO/WPU composites with different SGO contents.

Sample	Grinding Wheel Rotation Number/N	Mass Loss/mg
WPU-0	200	2.5
SGO/WPU-1	200	1.6
SGO/WPU-2	200	1.1
SGO/WPU-3	200	0.3
SGO/WPU-4	200	0.2
